# The Associations of Electronic Media Use With Sleep and Circadian Problems, Social, Emotional and Behavioral Difficulties in Adolescents

**DOI:** 10.3389/fpsyt.2022.892583

**Published:** 2022-06-09

**Authors:** Tim M. H. Li, Ngan Yin Chan, Chun-Tung Li, Jie Chen, Joey W. Y. Chan, Yaping Liu, Shirley Xin Li, Albert Martin Li, Jihui Zhang, Yun-Kwok Wing

**Affiliations:** ^1^Li Chiu Kong Family Sleep Assessment Unit, Department of Psychiatry, Faculty of Medicine, The Chinese University of Hong Kong, Shatin, Hong Kong SAR, China; ^2^Department of Psychology, The University of Hong Kong, Pokfulam, Hong Kong SAR, China; ^3^The State Key Laboratory of Brain and Cognitive Sciences, The University of Hong Kong, Pokfulam, Hong Kong SAR, China; ^4^Department of Pediatrics, Faculty of Medicine, The Chinese University of Hong Kong, Shatin, Hong Kong SAR, China; ^5^Guangdong Mental Health Center, Guangdong General Hospital and Guangdong Academy of Medical Sciences, Guangzhou, China

**Keywords:** adolescent, insomnia, eveningness, social jetlag, sleep deprivation, mental health, behavioral health, electronic media use

## Abstract

**Background:**

Electronic media use (EMU) becomes one of the most common activities in adolescents. The present study investigated the deleterious influence of excessive EMU and EMU before bedtime on social, emotional, and behavioral difficulties (SEBD) in adolescents. The role of sleep and circadian problems in mediating the association of EMU with SEBD was examined.

**Methods:**

A cross-sectional survey study was conducted with 3,455 adolescents (55.7% female, mean age = 14.8 ± 1.57 years, 36.6% monthly family income < HK$15,000) between December 2011 and March 2012 in Hong Kong. The associations of EMU with sleep and circadian problems and SEBD were analyzed using multiple binary logistic regression and path analysis. Sleep problems were measured by the Insomnia Severity Index and the reduced Horne and Östberg Morningness and Eveningness Questionnaire. Circadian problems were calculated based on established formulas. SEBD was measured using the Strengths and Difficulties Questionnaire. Participants' mental health status was assessed by the General Health Questionnaire.

**Results:**

A longer duration of EMU, excessive EMU (daily duration ≥ 2 h), and bedtime EMU (an hour before bedtime) were associated with the risk of sleep and circadian problems, poor mental health, and SEBD (*p* < 0.05). Insomnia, eveningness, social jetlag, and sleep deprivation were found to mediate the associations of EMU (including bedtime EMU of computers, electronic game consoles, phones, and televisions, together with excessive EMU of computers for leisure purposes and phones) with mental health and SEBD.

**Conclusions:**

The findings suggest the need for setting up guidelines and advocacy for education for appropriate EMU and intervention for the associated sleep and circadian problems to ameliorate EMU-related mental and behavioral health problems in adolescents.

## Introduction

Increasing evidence shows that electronic media use (EMU) is associated with adolescents' development and mental health (1). Adolescence is a critical transitional stage in physical, behavioral, and psychosocial development. Brain regions involved in extensive developmental transformation during adolescence are particularly impacted by EMU (2). Meta-analyses reported the association of excessive EMU with depressive symptoms and psychological distress in adolescents (3, 4). Excessive EMU (such as computer use, talking on the phone, and television viewing) may affect the holistic personal development of adolescents as it would replace other physical, social, and family activities. International public health guidelines have recommended that children should have no more than 2 h of EMU daily. When investigating the EMU behavior in adolescents, it is found that only one-third of adolescents worldwide have been able to adhere to the 2 h guideline suggestion (5, 6). Ostensibly, adolescents with excessive EMU are vulnerable to social, emotional, and behavioral difficulties (SEBD) (7, 8).

However, it is not simply excessive EMU that matters but also the timing of the use. In particular, EMU at night, especially before bedtime, could be even more problematic (9, 10). Exposure to light emitted from screen media (such as electronic game consoles, computers, and televisions) could suppress endogenous melatonin secretion with a consequent delay of circadian rhythm (11, 12). On the other hand, pubertal development also naturally shifts chronotype toward more eveningness (i.e., preferring later bedtime and wake time) (13, 14), which could perpetuate EMU at night. The two processes constitute a vicious cycle of EMU and reinforce sleep and circadian problems in adolescents. EMU before bedtime would increase the risk of insomnia (15), social jetlag (defined as the sleep time difference across weekdays and weekends) (16), and sleep deprivation (17). A number of studies suggest that adolescents with sleep and circadian problems were found to have a higher risk of excessive daytime sleepiness (EDS), social and behavioral problems, and poor mental health (18–23). Nevertheless, few studies have investigated the inter-relationship among EMU (especially on nighttime use), sleep and circadian problems, and SEBD (24–26).

Interestingly, different forms of electronic media might have differential effects on adolescents' sleep and mental health. While computer use, electronic gaming, and phone use all tend to be associated with sleep and circadian problems (15, 27, 28), television viewing was suggested to have a lower risk of psychological distress when compared to the use of other electronic devices (29, 30). It has been postulated that television viewing might provide educational benefits and family gathering opportunities that may facilitate adolescents' psychosocial development (30). Although computer usage is beneficial for the acquisition of knowledge and new ideas (via the Internet), exposure to age-inappropriate content may affect emotion and other internalizing problems in adolescents (31). Furthermore, the accompanying arousal during electronic gaming may exacerbate hyperactive behaviors (32).

The current study aimed to investigate the effects of excessive and bedtime EMU on sleep and circadian problems and SEBD. It was hypothesized that for different forms of electronic media, excessive and bedtime usage might have differential effects on the outcomes. The role of sleep and circadian problems in mediating the associations of excessive and bedtime EMU with SEBD were examined.

## Materials and Methods

### Study Design

This study was part of a large-scale, school-based sleep education program for adolescents in Hong Kong (Trial registry: ChiCTR-TRC-12002798) (33). Invitation letters were sent to all secondary schools in Hong Kong. Fifteen schools agreed to participate in the study (18, 33–35). Principals were contacted individually to further explain the details of the study. A set of paper-and-pencil self-administered questionnaires was delivered during school time. Participants provided written parental consent and individual assent, and they completed the questionnaires. The current study was based on the baseline cross-sectional data collected between December 2011 and March 2012, prior to the education program. The participants were students from grades 7 to 11 and aged 12 to 18 years. Those who reported a diagnosis of any psychiatric disorder(s) and/or having regular medication(s) in the past month were excluded. Ethical approval was obtained from the Joint CUHK-NTEC clinical research ethics committee (reference no: CRE-2011.249-T).

### Measures

The measures were classified into four domains: (a) demographic characteristics including age, gender, monthly family income (< HK$15,000 vs. ≥ HK$15,000; HK$7.8 = US$1), (b) SEBD, (c) sleep and circadian measures, and (d) excessive and bedtime EMU. Monthly family income < HK$15,000; HK$7.8 = US$1 was the lower income quartile in 2011 (36).

### Social, Emotional, and Behavioral Difficulties

The Strengths and Difficulties Questionnaire (SDQ) was used to measure the emotional and behavioral well-being of the adolescents (37). SDQ had five subscales to evaluate peer relationship problems, hyperactivity/inattention, emotional problems, conduct problems, and prosocial behavior. Each subscale contains 5 items with three-point Likert format (0 = not true to 2 = certainly true). The recommended cut-offs for identifying social and behavioral problems were adopted (http://www.sdqinfo.org/py/sdqinfo/c0.py). The Cronbach's alpha and the test-retest reliability of SDQ were 0.80 and 0.85, respectively (37).

The 12-item General Health Questionnaire (GHQ-12) was used to measure the overall mental health (38). In this study, GHQ-12 adopted a bimodal scoring method (0-0-1-1). The total score ranged from 0 to 12. A score ≥ 4 suggested poor mental health in Chinese adolescents (38). The Cronbach's alpha and the item-total correlation of GHQ-12 were 0.87 and 0.70, respectively.

The Pediatric Daytime Sleepiness Scale (PDSS) was used to measure the level of sleepiness in different situations (39). PDSS comprises 8 items with five-point Likert format (0 = never to 4 = always). The total score ranged from 0 to 32. A score > 19 indicated EDS in Chinese adolescents (sensitivity = 81%, specificity = 83%) (40). The Cronbach's alpha and the test-retest reliability of PDSS were 0.81 and 0.78, respectively.

### Sleep and Circadian Measures

The Insomnia Severity Index (ISI) was used to measure insomnia symptoms in the past 2 weeks (41). ISI includes 7 items with a five-point Likert format (0 = not at all to 4 = very much). The total score ranges from 0 to 28. A score ≥ 9 indicated clinically significant insomnia in Chinese adolescents (sensitivity = 87%, specificity = 75%) (41). The Cronbach's alpha and the test-retest reliability of ISI were 0.83 and 0.79, respectively.

The reduced Horne and Östberg Morningness and Eveningness Questionnaire (rMEQ) was used to measure chronotype preference (42). rMEQ consists of 5 items where the first 4 items were scored from 1 to 5 while the last item was scored from 0 to 6. The total score ranged from 4 to 26. Three classified types of chronotype were eveningness (score <12), intermediate-type (score 12–17), and morningness (score > 17). The Cronbach's alpha and the test-retest reliability of rMEQ were 0.70 and 0.77, respectively (43).

Other measures included sleep deprivation and social jetlag (44). The participants were asked “on weekdays/weekends, what is your bedtime/rise time usually?” Sleep duration was calculated as the difference between bedtime and rise time. Average sleep duration [(sleep duration during weekday × 5 + sleep duration during weekend × 2)/7] was calculated (19). According to a study relevant to this population (35), sleep deprivation was defined as having an average sleep duration of at least one standard deviation shorter than the mean sleep duration for that particular age. The difference between the bedtime on workdays and free days in participants ≥2 h was classified as social jetlag sleep-corrected (45).

### Electronic Media Use

The daily duration of EMU was assessed by asking participants how much time (hours and minutes) that they spent on (a) computer use for leisure purposes, (b) computer use for study purposes, (c) talking on the phone, and (d) television viewing. Daily duration ≥2 h was classified as excessive EMU (5, 6). The duration of EMU was the total number of hours spent on the electronic devices (a–d). Bedtime EMU was assessed using a yes/no checklist format: “What do you usually do an hour before bedtime at night?” Four items were included to assess whether participants would (I) spend time using computers, (II) play electronic games, (III) talk on the phone, and (IV) watch television an hour before bedtime (9, 10).

### Statistical Analysis

All statistical analyses were performed using statistical software R for Windows (R version 4.0.3). Descriptive statistics were presented as means and standard deviations for continuous variables, and as numbers and percentages for categorical variables. A *p*-value < 0.05 was considered statistically significant.

Multiple binary logistic regression was used to investigate: (1) the associations of the participants' demographic characteristics (predictors) with SEBD, sleep and circadian problems (including insomnia, eveningness, social jetlag, and sleep deprivation), and excessive and bedtime EMU (outcomes); (2) the effects of the duration of EMU (predictor) on sleep and circadian problems and SEBD (outcomes); (3) the effects of excessive and bedtime EMU of the studied electronic devices (predictor) on sleep and circadian problems (outcomes); (4) the effects of excessive and bedtime EMU of the studied electronic devices along with sleep and circadian problems (predictors) on SEBD (outcomes). The regression models were adjusted for age, gender, and monthly family income. Adjusted odds ratios (OR) with 95% confidence intervals (CI) were calculated as a measure of the strength of association.

**Figure 1:**
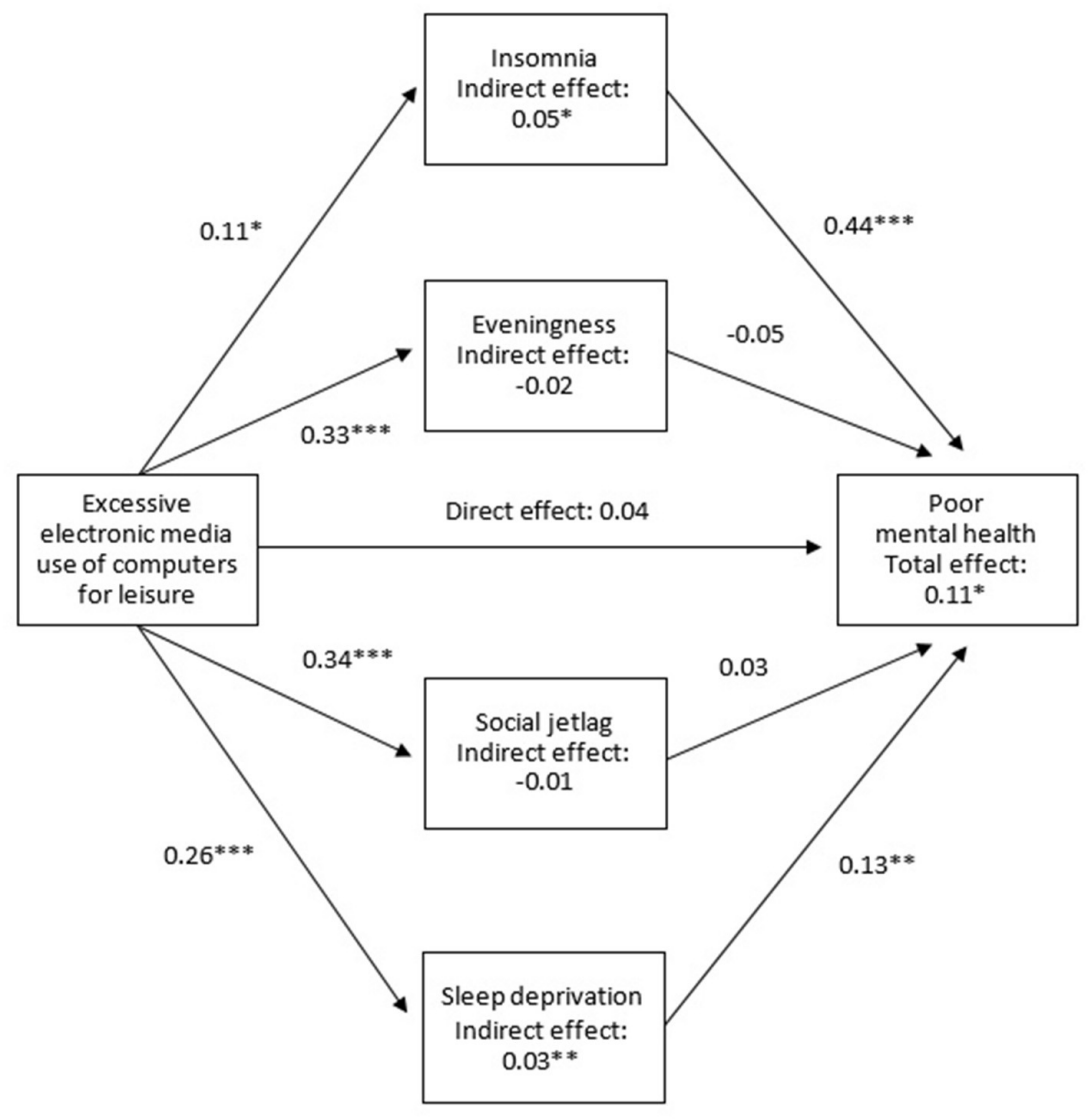
An example of a path model of the association among EMU, sleep and circadian problems, and SEDB (Insomnia measured by the Insomnia Severity Index; Eveningness measured by the reduced Horne and Östberg Morningness and Eveningness Questionnaire; Mental health measured by the 12-item General Health Questionnaire; Adjusted for age, gender, and monthly family income; * < 0.05, ** < 0.01, *** < 0.001).

Path analysis was conducted to investigate the association among EMU, sleep issues, and SEBD. Figure 1 depicts an example of a path model of the association among EMU, sleep and circadian problems, and SEBD. The indirect effects of EMU on SEBD through insomnia, eveningness, social jetlag, and sleep deprivation were calculated by the product of the coefficients approach. Age, gender, and monthly family income were included to adjust the path models. The models were estimated using diagonal weighted least squares because of the categorical data involved. Moreover, 5,000 bootstrap samples were used to infer significance of indirect effects. A path model was regarded as having a good fit to data if the comparative fit index (CFI) ≥ 0.95, root mean squared error of approximation (RMSEA) ≤ 0.05, and standardized root mean squared residual (SRMR) ≤ 0.08 (46).

## Results

### Sample Characteristics

A total of 8,236 students were eligible to participate in the study, and 63.4% (*n* = 5,219) returned the questionnaires. Among the respondents, 3,754 adolescents (71.9%) had valid data on demographic characteristics, social, emotional, and behavioral outcomes, sleep and circadian measures, as well as EMU information. Twenty-six and 273 adolescents who had a diagnosis of psychiatric disorder(s) and had been on regular medication(s) in the past month were excluded, respectively. The sample finally included 3,455 adolescents (55.7% female, mean age = 14.8 ± 1.57 years, 36.6% monthly family income < HK$15,000).

Table 1 shows the associations between the studied variables and demographics. In the sample, 17.3–29.9% of the participants had SEBD. More males had conduct problems, peer relationship problems, and prosocial behavior problems, while more females had emotional symptoms, poor mental health, and EDS. Younger participants were more likely to have prosocial behavioral problems, but less likely to have poor mental health and EDS. Monthly family income < HK$15,000 was associated with peer relationship problems and prosocial behavior problems.

**Table 1:** Associations between the studied variables and demographics (including age, gender, and family income).

**Variables**		**Age**		**Being female**		**Monthly family income ≤HK$15,000**	
	***n* (%)**	**Adjusted OR**	***p***	**Adjusted OR**	***p***	**Adjusted OR**	***p***
**SEBD**							
Poor mental health	581 (16.8)	1.13 (1.07, 1.20)***	<0.001	1.69 (1.40, 2.04)***	<0.001	1.17 (0.97, 1.40)	0.10
Emotional symptoms	597 (17.3)	1.03 (0.97, 1.09)	0.30	2.50 (2.06, 3.05)***	<0.001	0.98 (0.82, 1.18)	0.87
Peer relationship problems	968 (28.0)	0.96 (0.91, 1.01)	0.09	0.59 (0.51, 0.68)***	<0.001	1.35 (1.16, 1.58)***	<0.001
Prosocial behavior problems	969 (28.0)	0.93 (0.89, 0.98)**	0.005	0.54 (0.47, 0.63)***	<0.001	1.42 (1.22, 1.66)***	<0.001
Conduct problems	701 (20.3)	1.01 (0.96, 1.07)	0.60	0.77 (0.65, 0.91)**	0.002	1.01 (0.85, 1.20)	0.93
hyperactivity/inattention	646 (18.7)	1.02 (0.96, 1.07)	0.58	1.11 (0.93, 1.32)	0.24	1.01 (0.85, 1.21)	0.89
EDS	1032 (29.9)	1.16 (1.10, 1.21)***	<0.001	1.81 (1.55, 2.11)***	<0.001	1.03 (0.89, 1.21)	0.66
**Sleep and circadian problems**							
Insomnia	1225 (35.5)	1.19 (1.14, 1.25)***	<0.001	1.45 (1.26, 1.68)***	<0.001	1.34 (1.16, 1.55)***	<0.001
Eveningness	766 (22.2)	1.16 (1.10, 1.22)***	<0.001	1.26 (1.07, 1.48)**	0.007	0.97 (0.82, 1.15)	0.76
Social jetlag	877 (25.4)	1.15 (1.10, 1.21)***	<0.001	1.05 (0.90, 1.23)	0.55	1.17 (1.00, 1.38)*	0.04
Sleep deprivation	520 (15.1)	1.02 (0.96, 1.09)	0.45	1.42 (1.17, 1.72)***	<0.001	0.80 (0.66, 0.98)*	0.03
**Excessive EMU**							
Computer for leisure	1411 (40.8)	1.13 (1.08, 1.18)***	<0.001	0.84 (0.74, 0.97)*	0.02	1.28 (1.11, 1.47)***	<0.001
Computer for study	444 (12.9)	1.20 (1.13, 1.28)***	<0.001	1.42 (1.15, 1.75)**	0.001	1.06 (0.86, 1.30)	0.59
Phone	236 (6.8)	1.06 (0.98, 1.15)	0.16	1.06 (0.81, 1.39)	0.67	1.13 (0.86, 1.49)	0.36
Television	1516 (43.9)	0.92 (0.88, 0.96)***	<0.001	1.07 (0.93, 1.23)	0.33	1.34 (1.17, 1.55)***	<0.001
**Bedtime EMU**							
Computer	2388 (69.1)	1.21 (1.16, 1.27)***	<0.001	1.15 (0.99, 1.33)	0.07	1.00 (0.86, 1.17)	>0.99
Electronic game console	698 (20.2)	1.03 (0.98, 1.09)	0.24	0.34 (0.29, 0.41)***	<0.001	0.85 (0.71, 1.02)	0.08
Phone	812 (23.5)	1.04 (0.99, 1.10)	0.10	1.37 (1.16, 1.61)***	<0.001	0.95 (0.80, 1.12)	0.52
Television	2201 (63.7)	0.84 (0.80, 0.88)***	<0.001	1.01 (0.88, 1.16)	0.90	1.21 (1.05, 1.41)*	0.01

Mental health measured by the 12-item General Health Questionnaire; SEBD, Social, emotional, and behavioral difficulties (measured by the Strengths and Difficulties Questionnaire); EDS, Excessive daytime sleepiness (measured by the Pediatric Daytime Sleepiness Scale); Insomnia measured by the Insomnia Severity Index; Eveningness measured by the reduced Horne and Östberg Morningness and Eveningness Questionnaire; EMU, Electronic media use; * < 0.05, ** < 0.01, *** < 0.001.

Sleep and circadian problems were reported in 15.1–35.5% of the overall sample. More females had insomnia, eveningness, social jetlag, and sleep deprivation. Older participants were more likely to have insomnia, eveningness, and social jetlag. Monthly family income < HK$15,000 was associated with insomnia and social jetlag in the adolescent subjects.

The duration of EMU was 4.9 ± 3.02 h. Excessive EMU was reported in 6.8–43.9% of the overall sample, while 20.2–69.1% of the participants had bedtime EMU. More males had excessive EMU of computer for leisure and bedtime EMU for electronic game playing, while more females had excessive EMU of computer for study. Younger participants were more likely to have excessive and bedtime EMU of television, while older participants were more likely to have excessive and bedtime EMU of computers. Monthly family income < HK$15,000 was associated with excessive and bedtime EMU of television and excessive EMU of computer for leisure.

### The Effect of the Duration of EMU on Sleep and Circadian Problems and SEBD

Table 2 reveals the effects of the duration of EMU on SEBD and sleep and circadian problems. For sleep and circadian problems, every 1-h increase in the duration of EMU was associated with 9, 10, and 8% of the increased odds of eveningness, social jetlag, and sleep deprivation, respectively. For SEBD, every 1-h increase in the duration of EMU was associated with a 3–5% increase in the risk of poor mental health, emotional symptoms, prosocial behavior problems, conduct problems, hyperactivity/inattention, and EDS. There were no significant association of the duration of EMU with peer relationship problems and insomnia.

**Table 2:** The effects of the duration of EMU on SEBD and sleep and circadian problems.

**Variables**	**Duration of EMU**	
	**Adjusted OR**	***p***
**SEBD**		
Poor mental health	1.03 (1.00, 1.06)*	0.04
Emotional symptoms	1.04 (1.01, 1.07)*	0.01
Peer relationship problems	0.99 (0.96, 1.01)	0.32
Prosocial behavior problems	1.03 (1.01, 1.06)*	0.02
Conduct problems	1.03 (1.01, 1.06)*	0.01
hyperactivity/inattention	1.03 (1.00, 1.06)*	0.05
EDS	1.05 (1.03, 1.08)***	<0.001
**Sleep and circadian problems**		
Insomnia	1.01 (0.99, 1.03)	0.36
Eveningness	1.09 (1.07, 1.12)***	<0.001
Social jetlag	1.10 (1.07, 1.12)***	<0.001
Sleep deprivation	1.08 (1.05, 1.11)***	<0.001

Mental health measured by the 12-item General Health Questionnaire; SEBD, Social, emotional, and behavioral difficulties (measured by the Strengths and Difficulties Questionnaire); EDS, Excessive daytime sleepiness (measured by the Pediatric Daytime Sleepiness Scale); Insomnia measured by the Insomnia Severity Index; Eveningness measured by the reduced Horne and Östberg Morningness and Eveningness Questionnaire; EMU, Electronic media use; Adjusted for age, gender, and monthly family income; * < 0.05 and *** < 0.001.

### Comparison of the Effects of Excessive and Bedtime EMU on Sleep and Circadian Problems

The four sleep and circadian problems were all associated with each other (OR = 1.19–2.49, *p* < 0.05). Table 3 reveals the comparison of the effects of excessive and bedtime EMU of different electronic devices on each problem. Excessive EMU of computers for leisure was associated with a 59, 63, and 42% increase in the risk of eveningness, social jetlag, and sleep deprivation, respectively. Excessive EMU of computers for study was associated with 39% of reduced odds of social jetlag. Participants with excessive EMU of phones were 50, 89, and 66% more likely to experience eveningness, social jetlag, and sleep deprivation, respectively, than those without excessive EMU of the phone. There were no significant associations between excessive EMU of televisions and the sleep and circadian problems.

**Table 3:** Comparison of the effects of excessive and bedtime EMU of different electronic devices on sleep and circadian problems.

**Predictors**	**Insomnia**		**Eveningess**		**Social jetlag**		**Sleep deprivation**	
	**Adjusted OR**	***p***	**Adjusted OR**	***p***	**Adjusted OR**	***p***	**Adjusted OR**	***p***
**Excessive EMU**								
Computer for leisure	1.16 (1.00, 1.35)	0.05	1.59 (1.34, 1.89)***	<0.001	1.63 (1.38, 1.92)***	<0.001	1.42 (1.16, 1.74)***	<0.001
Computer for study	1.00 (0.81, 1.24)	0.97	0.95 (0.74, 1.21)	0.68	0.61 (0.47, 0.78)***	<0.001	1.23 (0.94, 1.60)	0.13
Phone	1.22 (0.92, 1.63)	0.17	1.50 (1.10, 2.02)**	0.009	1.89 (1.41, 2.52)***	<0.001	1.66 (1.18, 2.31)**	0.003
Television	0.93 (0.80, 1.08)	0.34	0.94 (0.79, 1.12)	0.50	1.07 (0.90, 1.27)	0.45	0.91 (0.74, 1.11)	0.34
**Bedtime EMU**								
Computer	1.15 (0.98, 1.36)	0.09	1.27 (1.05, 1.55)*	0.02	1.27 (1.05, 1.53)*	0.01	1.32 (1.05, 1.67)*	0.02
Electronic game console	0.97 (0.81, 1.17)	0.78	1.56 (1.27, 1.91)***	<0.001	1.55 (1.27, 1.87)***	<0.001	1.28 (1.01, 1.62)*	0.04
Phone	1.25 (1.05, 1.48)*	0.01	1.49 (1.23, 1.81)***	<0.001	1.62 (1.35, 1.95)***	<0.001	1.35 (1.08, 1.68)**	0.009
Television	0.77 (0.66, 0.90)***	<0.001	0.69 (0.58, 0.82)***	<0.001	1.04 (0.87, 1.24)	0.68	0.59 (0.48, 0.73)***	<0.001

Insomnia measured by the Insomnia Severity Index; Eveningness measured by the reduced Horne and Östberg Morningness and Eveningness Questionnaire; EMU, Electronic media use; Adjusted for age, gender, and monthly family income; * < 0.05, ** < 0.01, *** < 0.001.

Participants with bedtime EMU of computers were 27, 27, and 32% more likely to experience eveningness, social jetlag, and sleep deprivation, respectively, than those without bedtime EMU of computers. Bedtime EMU of electronic game consoles was associated with 56, 55, and 28% of the increased likelihood of eveningness, social jetlag, and sleep deprivation, respectively. Participants with bedtime EMU of phones were 25, 49, 62, and 35% more likely to experience insomnia, eveningness, social jetlag, and sleep deprivation, respectively than those without bedtime EMU of phones. Bedtime EMU of televisions was associated with 23, 31, and 41% of the reduced odds of insomnia, eveningness, and sleep deprivation, respectively.

### Comparison of the Effects of Excessive and Bedtime EMU, Sleep and Circadian Problems on SEBD

Table 4 compares the effects of excessive and bedtime EMU of different electronic devices and sleep and circadian problems on each SEBD. Excessive EMU of computers for leisure was associated with a 21 and 23% increase in the risk of prosocial behavior problems and EDS, respectively. Excessive EMU of computers for study was associated with a 30% increase in the risk of peer relationship problems. Participants with excessive EMU of phones were 43 and 44% more likely to have prosocial behavior problems and hyperactivity/inattention, respectively than those without excessive EMU of phones. There were no associations of excessive EMU of television with SEBD.

**Table 4:** Comparison of the effects of excessive and bedtime EMU of different electronic devices and sleep and circadian problems on SEBD.

**Predictors**	**Poor mental health**		**Emotional symptoms**		**Peer relationship problems**		**Prosocial behavior problems**		**Conduct problems**		**hyperactivity/inattention**		**EDS**	
	**Adjusted OR**	***p***	**Adjusted OR**	***p***	**Adjusted OR**	***p***	**Adjusted OR**	***p***	**Adjusted OR**	***p***	**Adjusted OR**	***p***	**Adjusted OR**	***p***
**Excessive EMU**
Computer for leisure	1.06 (0.87, 1.30)	0.54	1.00 (0.82, 1.23)	0.99	0.90 (0.76, 1.06)	0.21	1.21 (1.03, 1.42)*	0.02	1.07 (0.89, 1.28)	0.49	1.09 (0.90, 1.31)	0.39	1.23 (1.04, 1.46)*	0.02
Computer for study	1.26 (0.96, 1.64)	0.09	1.03 (0.78, 1.36)	0.81	1.30 (1.03, 1.63)*	0.02	0.99 (0.78, 1.24)	0.92	0.92 (0.70, 1.19)	0.52	0.81 (0.61, 1.07)	0.14	1.10 (0.87, 1.40)	0.42
Phone	1.26 (0.88, 1.78)	0.20	1.18 (0.82, 1.68)	0.37	0.88 (0.63, 1.21)	0.43	1.43 (1.04, 1.94)*	0.02	1.26 (0.91, 1.72)	0.17	1.44 (1.03, 1.99)*	0.03	1.14 (0.83, 1.56)	0.42
Television	1.06 (0.87, 1.30)	0.57	1.13 (0.92, 1.38)	0.25	1.06 (0.90, 1.25)	0.49	1.02 (0.87, 1.20)	0.81	0.95 (0.79, 1.15)	0.62	1.04 (0.86, 1.26)	0.65	1.05 (0.88, 1.25)	0.60
**Bedtime EMU**
Computer	0.97 (0.78, 1.21)	0.77	1.03 (0.82, 1.28)	0.82	0.78 (0.66, 0.92)**	0.004	0.88 (0.75, 1.05)	0.16	0.94 (0.77, 1.14)	0.53	0.98 (0.80, 1.21)	0.87	1.26 (1.05, 1.53)*	0.02
Electronic game console	0.96 (0.75, 1.23)	0.77	0.97 (0.74, 1.24)	0.79	1.25 (1.03, 1.52)*	0.02	1.40 (1.16, 1.70)***	<0.001	1.10 (0.89, 1.36)	0.38	1.24 (0.99, 1.54)	0.06	1.06 (0.86, 1.31)	0.57
Phone	1.34 (1.07, 1.67)*	0.01	1.14 (0.90, 1.43)	0.27	0.88 (0.72, 1.07)	0.20	0.66 (0.54, 0.81)***	<0.001	1.37 (1.12, 1.68)**	0.002	1.08 (0.87, 1.34)	0.47	1.34 (1.11, 1.63)**	0.003
Television	0.81 (0.66, 0.99)*	0.04	0.78 (0.63, 0.96)*	0.02	0.80 (0.68, 0.95)**	0.009	0.92 (0.78, 1.09)	0.36	0.87 (0.72, 1.05)	0.15	0.86 (0.71, 1.05)	0.14	0.79 (0.67, 0.95)*	0.01
**Sleep and circadian problems**														
Insomnia	3.86 (3.18, 4.69)***	<0.001	4.57 (3.76, 5.58)***	<0.001	1.77 (1.51, 2.08)***	<0.001	1.42 (1.21, 1.67)***	<0.001	2.35 (1.97, 2.80)***	<0.001	2.64 (2.20, 3.17)***	<0.001	3.92 (3.34, 4.62)***	<0.001
Eveningness	0.97 (0.78, 1.22)	0.82	1.20 (0.96, 1.50)	0.10	1.01 (0.83, 1.22)	0.95	1.07 (0.89, 1.30)	0.46	1.38 (1.13, 1.68)**	0.002	1.41 (1.14, 1.73)**	0.001	2.42 (2.01, 2.92)***	<0.001
Social jetlag	1.11 (0.89, 1.37)	0.36	1.22 (0.98, 1.51)	0.07	1.09 (0.90, 1.30)	0.38	1.13 (0.94, 1.35)	0.20	1.31 (1.08, 1.59)**	0.007	1.06 (0.86, 1.30)	0.58	1.06 (0.87, 1.27)	0.57
Sleep deprivation	1.52 (1.19, 1.93)***	<0.001	1.60 (1.25, 2.03)***	<0.001	0.91 (0.72, 1.13)	0.38	0.87 (0.69, 1.08)	0.21	1.00 (0.79, 1.27)	0.98	1.41 (1.12, 1.77)**	0.004	1.25 (1.00, 1.55)*	0.05

Mental health measured by the 12-item General Health Questionnaire; SEBD, Social, emotional, and behavioral difficulties (measured by the Strengths and Difficulties Questionnaire); EDS, Excessive daytime sleepiness (measured by the Pediatric Daytime Sleepiness Scale); Insomnia measured by the Insomnia Severity Index; Eveningness measured by the reduced Horne and Östberg Morningness and Eveningness Questionnaire; EMU, Electronic media use; Adjusted for age, gender, and monthly family income; * < 0.05, ** < 0.01, *** < 0.001.

Bedtime EMU of computers was associated with 26% of the increased odds of EDS but 22% of reduced odds of peer relationship problems. Bedtime EMU of electronic game consoles was associated with 25 and 40% of the increased risk of peer relationship problems and prosocial behavior problems, respectively. Participants with bedtime EMU of phones were 34, 66, 37, and 34% more likely to have poor mental health, prosocial behavior problems, conduct problems, and EDS, respectively, than those without bedtime EMU of phones. Bedtime EMU of televisions was associated with 19, 20, and 21% of reduced likelihood of emotional symptoms, peer relationship problems, and EDS, respectively.

Participants with insomnia were 1.42 to 4.57 times more likely to have SEBD than those without insomnia. Participants with eveningness were 1.38, 1.41, and 2.42 times more likely to have conduct problems, hyperactivity/inattention, and EDS than those without eveningness, respectively. Social jetlag was associated with a 31% of increased risk of conduct problems. Sleep deprivation was associated with 52, 60, 41, and 25% of the increased odds of poor mental health, emotional symptoms, hyperactivity/inattention, and EDS, respectively.

### The Effect of Sleep and Circadian Problems in the Associations of EMU With SEBD

Table 5 demonstrates the significant effects of sleep and circadian problems in the associations of EMU with SEBD in path models. All the models had satisfactory goodness-of-fit indices. Overall, insomnia, eveningness, social jetlag, and sleep deprivation mediated the effect of excessive and bedtime EMU on SEBD. In particular, insomnia mediated the associations of excessive EMU of computers for leisure and phones, along with bedtime EMU of computers, phones, and televisions on all kinds of SEBD. For emotional difficulties, sleep deprivation mediated the associations of excessive EMU of computers for leisure and phones, along with bedtime EMU of phones and televisions on poor mental health and emotional symptoms. For behavioral difficulties, social jetlag mediated the associations of excessive and bedtime EMU of phones on conduct problems. Eveningness mediated the associations of excessive EMU of computers for leisure and phones on externalizing difficulties (including both hyperactivity/inattention and conduct problems). Eveningness also mediated the associations of excessive EMU of computers for leisure and phones, along with bedtime EMU of computers, electronic game consoles, phones, and televisions on EDS. While most EMU was associated with increased sleep and circadian problems and SEBD, it is interesting to note that bedtime EMU of television viewing improved insomnia, eveningness, and sleep deprivation with lesser SEBD problems.

**Table 5:** The significant effects of sleep and circadian problems in the associations of excessive and bedtime EMU with social, emotional, and behavioral difficulties.

**Predictors**	**Outcomes**	**Direct effect**	***p***	**Indirect effect (Insomnia)**	***p***	**Indirect effect (Eveningness)**	***p***	**Indirect effect (Social jetlag)**	***p***	**Indirect effect (Sleep deprivation)**	***p***	**Total effect**	***p***
Excessive EMU of computers for leisure	Poor mental health	0.04	0.43	0.05*	0.02	−0.02	0.22	0.01	0.48	0.03**	0.009	0.11*	0.03
Excessive EMU of phones	Poor mental health	0.18	0.06	0.09*	0.02	−0.02	0.21	0.01	0.55	0.05*	0.01	0.32***	<0.001
Bedtime EMU of phones	Poor mental health	0.14*	0.02	0.07**	0.004	−0.02	0.21	0.01	0.61	0.03*	0.02	0.23***	<0.001
Bedtime EMU of televisions	Poor mental health	−0.06	0.29	−0.07***	<0.001	0.01	0.23	0.00	0.55	−0.03**	0.007	−0.15**	0.004
Excessive EMU of computers for leisure	Emotional symptoms	0.00	0.98	0.05*	0.02	0.00	0.89	0.01	0.25	0.03*	0.01	0.10*	0.04
Excessive EMU of phones	Emotional symptoms	0.08	0.39	0.10*	0.02	0.00	0.89	0.02	0.29	0.05*	0.01	0.26**	0.005
Bedtime EMU of phones	Emotional symptoms	0.05	0.42	0.07**	0.003	0.00	0.91	0.02	0.31	0.03*	0.02	0.17**	0.004
Bedtime EMU of televisions	Emotional symptoms	−0.06	0.23	−0.08***	<0.001	0.00	0.92	0.00	0.42	−0.03**	0.010	−0.17**	0.001
Bedtime EMU of computers	Peer relationship problems	−0.16**	0.002	0.03*	0.02	0.00	0.79	0.01	0.60	−0.01	0.36	−0.14**	0.004
Bedtime EMU of televisions	Peer relationship problems	−0.12*	0.01	−0.03**	0.002	0.00	0.70	0.00	0.72	0.01	0.23	−0.14**	0.004
Excessive EMU of computers for leisure	Prosocial behavior problems	0.12*	0.01	0.01*	0.04	0.01	0.59	0.01	0.26	−0.02	0.17	0.14**	0.003
Bedtime EMU of phones	Prosocial behavior problems	−0.22***	0.00	0.02*	0.02	0.01	0.44	0.02	0.10	−0.01	0.24	−0.17**	0.001
Excessive EMU of phones	Conduct problems	0.17	0.08	0.06*	0.02	0.04*	0.048	0.05*	0.03	−0.01	0.67	0.31***	<0.001
Bedtime EMU of televisions	Conduct problems	−0.05	0.31	−0.05**	0.001	−0.02	0.05	0.01	0.27	0.01	0.62	−0.11*	0.04
Bedtime EMU of phones	Conduct problems	0.16**	0.006	0.05**	0.004	0.03	0.05	0.03*	0.04	0.00	0.66	0.26***	<0.001
Excessive EMU of computers for leisure	Hyperactivity/inattention	0.03	0.50	0.04*	0.02	0.03*	0.04	0.00	0.81	0.02	0.05	0.12*	0.01
Excessive EMU of phones	Hyperactivity/inattention	0.20*	0.03	0.07*	0.02	0.04	0.06	0.00	0.92	0.04	0.05	0.34***	<0.001
Bedtime EMU of phones	Hyperactivity/inattention	0.05	0.36	0.05**	0.004	0.03*	0.04	0.00	0.84	0.02	0.05	0.15**	0.007
Bedtime EMU of televisions	Hyperactivity/inattention	−0.03	0.58	−0.05***	<0.001	−0.02	0.06	0.00	0.78	−0.02	0.05	−0.12*	0.02
Excessive EMU of computers for leisure	EDS	0.10*	0.03	0.05*	0.01	0.09***	<0.001	−0.01	0.65	0.01	0.36	0.24***	<0.001
Excessive EMU of phones	EDS	0.12	0.14	0.09*	0.02	0.12***	<0.001	−0.01	0.70	0.02	0.34	0.34***	<0.001
Bedtime EMU of computers	EDS	0.12*	0.01	0.05*	0.02	0.07***	<0.001	0.00	0.68	0.01	0.39	0.25***	<0.001
Bedtime EMU of electronic game consoles	EDS	0.04	0.50	0.01	0.80	0.09***	<0.001	0.00	0.73	0.01	0.37	0.14*	0.02
Bedtime EMU of phones	EDS	0.13**	0.009	0.07**	0.003	0.08***	<0.001	−0.01	0.59	0.01	0.34	0.28***	<0.001
Bedtime EMU of televisions	EDS	−0.04	0.33	−0.07***	<0.001	−0.05***	<0.001	0.00	0.84	−0.01	0.36	−0.18***	<0.001

Mental health measured by the 12-item General Health Questionnaire; SEBD, Social, emotional, and behavioral difficulties (measured by the Strengths and Difficulties Questionnaire); EDS, Excessive daytime sleepiness (measured by the Pediatric Daytime Sleepiness Scale); Insomnia measured by the Insomnia Severity Index; Eveningness measured by the reduced Horne and Östberg Morningness and Eveningness Questionnaire; EMU, Electronic media use; Adjusted for age, gender, and monthly family income; * < 0.05, ** < 0.01, *** < 0.001.

## Discussion

The present study investigated the relationships among EMU, sleep and circadian problems, and SEBD in adolescents. In line with previous research (9, 10), the study highlights that bedtime EMU of computers, electronic game consoles, and phones contributed to the risk of sleep and circadian problems. This could be explained by nocturnal exposure to screen light, especially blue light, which further increases arousal and suppresses melatonin levels with a consequent delay of circadian rhythm, leading to insomnia, eveningness, social jetlag, and sleep deprivation (11). The findings also reveal that excessive EMU of computers for leisure and phones emerged as risk factors for sleep and circadian problems. It is suspected that interactive and stimulating EMU may increase the level of circulating catecholamines and hence delay sleep onset (47).

In addition, EMU was related to adolescents' SEBD. EMU throughout the day and at night concurrently contributes to the risk of poor mental health in adolescents (7). Different forms of electronic media could have different effects on adolescents' SEBD. Bedtime EMU of electronic game consoles, for example, was associated significantly with peer relationship problems and prosocial behavior problems, and marginally with hyperactivity/inattention in the study, which echoes the suggestion that the heightened arousal during electronic gaming exacerbates hyperactive behaviors and related psychosocial problems (32). The arousal can be triggered by exposure to online game violence and cyberbullying with the aggressive affective state (48). Excessive and bedtime EMU of phones were also risk factors for poor mental health, conduct problems, hyperactivity/inattention, and EDS. Adolescents with excessive EMU of phones may rely on phone calls, text messages, and social media networks for communication, which potentially reduces face-to-face interaction with family and peers (26). On the other hand, passive bedtime EMU of televisions was found, interestingly, to be a protective factor for peer relationships. This may be construed as having the benefits of educational/cultural television programs and family time during television viewing for better psychosocial development (30). Passive EMU involves only information consumption so adolescents can still interact with others during television viewing. As reported by previous studies, EMU of television viewing was linked to depressive symptoms and psychological distress, probably reflecting a sedentary lifestyle (29, 30), which was not found in the current study. On the contrary, bedtime EMU of televisions reduced emotional symptoms. Further investigation is required to corroborate the influence of different types of EMU including television viewing on adolescents' mental health and psychosocial development.

In this study, the data was collected in 2011 and did not cover time spent on smartphones and tablets, which are commonly used by adolescents nowadays. While caution should be taken when interpreting adolescents' EMU pattern, the findings are consistent with recent studies that sleep and circadian problems are linked to developmental problems in adolescents (18–21) and the use of various electronic devices contributed to SEBD (29, 30). The data is useful to further investigate the inter-relationship among EMU, sleep and circadian problems, and SEBD that few large-scale studies have examined. The hypothesis that EMU was indirectly related to SEBD through sleep and circadian problems (24–26) is supported by the findings that insomnia, eveningness, social jetlag, and sleep deprivation mediated the effect of excessive and bedtime EMU on SEBD. In particular, insomnia independently mediated the associations of excessive and bedtime EMU with social and behavioral difficulties including peer relationship problems, prosocial behavioral problems, conduct problems, and hyperactivity/inattention. The mediating role of insomnia can be related to the physiological hyperarousal and cognitive-emotional dysregulation that may lead to psychopathologies, behavioral disturbances, and maladaptive fear responses (18–20). In turn, the fears and arousal will further predispose adolescents to be more vulnerable to negative experiences. Insomnia and sleep deprivation also jointly mediated the relationships between EMU and emotional difficulties, including poor mental health and emotional symptoms. Previous research identified the adverse effect of sleep deprivation on adolescents' emotional processes such as sleep-dependent emotional regulation, emotional reactivity, and emotional memory processing (22). In addition, eveningness mediated the relationship between EMU and EDS, affecting adolescents' daily functioning. It is suggested that evening-type adolescents tend to be less adaptive to their daily routines and academic performance compared to their morning-oriented counterparts (13, 14).

There were several limitations to this study. First, the study was a cross-sectional study that could not infer any causal relationship among EMU, sleep and circadian problems, and SEBD. A prospective longitudinal study should be conducted to confirm that adolescents' SEBD is affected directly by EMU and indirectly through sleep and circadian problems over time. Second, the response rate was only modest, albeit a large-scale sample was collected. The sample was collected from 15 different local schools to reduce selection bias. Future research can adopt random sampling to collect a more representative sample. Third, the self-construct questionnaires can be modified to improve the validity. For example, the calculation of social jetlag and sleep deprivation was based on rise time and bedtime, which could underestimate the problems. The daily EMU variable did not take into consideration usage on weekdays vs. weekends/holidays, which could be quite different. Bedtime EMU was assessed using a yes/no checklist format, while the time spent was not measured, which limited the investigation of the quantity of bedtime EMU. Finally, the data on EMU, sleep and circadian problems, and SEBD were based on retrospective self-report measures and were therefore subjected to recall bias. It is believed that studies using more objective measures are warranted. Nevertheless, there was scarce evidence to suggest a more reliable measure of EMU other than self-report (8). Future research is needed to develop novel technical tools, for example, smart glasses measuring real-time EMU exposure more accurately at eye level (49). Apart from the duration and types, the content of EMU is also worth investigating.

The study provides important research and clinical implications. Existing guidelines are mainly used for preventing excessive EMU in children (5, 6). In Hong Kong, the government proposes a limit of recreational screen time to be no more than 2 h a day for children under the age of 12 (50). However, there is no such restriction for adolescents. The study demonstrates that excessive EMU can affect adolescents pervasively. The findings advocate the need for a public health guideline to set a healthy daily electronic media usage limit and EMU restraint before bedtime for adolescents. Given that the mediating effect of sleep and circadian problems on the associations of EMU with SEBD, there should be additional and appropriate intervention and prevention strategies to tackle sleep and circadian problems. Apart from advice and education in restricting EMU, the use of cognitive-behavioral therapy for insomnia, bright light therapy, sleep education, and in some clinical cases, judicious use of medications such as melatonin may be required for intervening insomnia, eveningness, social jetlag, and sleep deprivation to ameliorate EMU-related mental and behavioral health problems.

## Conclusion

The present study demonstrates the relationship among EMU, sleep and circadian problems, and SEBD in adolescents. Insomnia, eveningness, social jetlag, and sleep deprivation seemed to mediate the associations of excessive and bedtime EMU with SEBD. In addition to appropriate education for limiting excessive and bedtime EMU, these findings highlight the need for intervening sleep and circadian problems to ameliorate EMU-related mental and behavioral health problems in adolescents.

## Data Availability Statement

The raw data supporting the conclusions of this article will be made available by the authors, without undue reservation.

## Ethics Statement

The studies involving human participants were reviewed and approved by the Joint CUHK-NTEC Clinical Research Ethics Committee (reference no: CRE-2011.249-T). Written informed consent to participate in this study was provided by the participants' legal guardian/next of kin.

## Author Contributions

NC, SL, and Y-KW designed the study. TL performed the literature search, conducted data analysis, and wrote the first draft of the manuscript. C-TL, JC, JYC, YL, AL, and JZ provided critical comments for revising the manuscript. All authors contributed to the article and approved the submitted version.

## Funding

This work was supported by the Public Policy Research of University Grants Committee (Reference number: CUHK4012-PPR-11), Hong Kong SAR, China.

## Conflict of Interest

Y-KW received a consultation fee for delivering a lecture from Eisai Co., Ltd. The remaining authors declare that the research was conducted in the absence of any commercial or financial relationships that could be construed as a potential conflict of interest.

## Publisher's Note

All claims expressed in this article are solely those of the authors and do not necessarily represent those of their affiliated organizations, or those of the publisher, the editors and the reviewers. Any product that may be evaluated in this article, or claim that may be made by its manufacturer, is not guaranteed or endorsed by the publisher.
